# Risk characterisation and methods of improving practice for municipal waste management in disaster situations: A case study in Qom Province, Iran

**DOI:** 10.4102/jamba.v14i1.1151

**Published:** 2022-06-15

**Authors:** Yadollah Ghafuri, Alireza Koohpaei

**Affiliations:** 1 Research Center for Environmental Pollutants, Qom University of Medical Sciences, Qom, Iran; 2 Department of Occupational Health, Work Health Research Centre, Qom University of Medical Sciences, Qom, Iran

**Keywords:** Qom, waste, management, disaster, risk

## Abstract

Developing strategies to control environmental challenges and various aspects of health consequences of waste management is one of the major programs in metropolitan management. The main purpose of this study is to examine the level of preparedness, having a contingency plan and the emergency response ability to waste management in disaster situations. A descriptive study was designed in two phases: in the first phase of the study, composition, quantity and characteristics of municipal solid waste in the disaster were estimated, and by using DotMapper software, temporary waste sites for disaster situations (map waste) were determined. In the second phase of the study, the preliminary hazard analysis (PHA) to identify the initial events and risk analysis in the municipal waste management system was considered. Results show that more than 50% of the generated waste in the disaster is allocated to construction waste resulting from the destruction and more than 30% to recyclable items (metals, glass, plastic), and a very small part of about 1% of biodegradable waste was determined. Twenty points were designated as temporary sites for municipal waste in a disaster situation. Results of risk analysis in the disaster were described that for three events containing lack of temporary waste storage sites, lack of identification and determination of hazardous waste production centres and lack of waste management training programme in emergency situations were in the red range. Developing the necessary strategies to control environmental challenges and various aspects of health outcomes in waste management is one of the basic programmes in metropolitan management.

## Introduction

Unexpected events are among the most important issues that affect governments and nations every year, inflicting heavy casualties and financial losses on them. In the last 20 years, unforeseen events have killed nearly 10 million people around the world (Alexander 2015). Experience has shown that acting responsibly in the event of a disaster is not just about having enough information but also about understanding what is appropriate for the threat. Accordingly, service providers in the field of urban management, as the main organisations involved in the occurrence of unforeseen events, need an action plan to deal with disasters and crises (Brown, Milke & Seville 2011). In the municipal waste management system, due to the extent and related areas, the subject of proactive approach in the field of emergencies and disasters is one of the basic aspects of urban waste management (Hayes et al. 2021). Moral and psychological problems related to debris and destroyed buildings, accumulation of debris that has become a collection site for other waste in different areas, obstruction and disruption of the municipal service waste collection fleet, entry of hazardous waste and especially uncontrolled accumulation of medical waste in medical centres and clinics are one of the topics that should be considered in waste management in emergencies (Shuai et al. 2009). In recent years, the issue of waste management in disasters has become very important, and in most developed countries, various strategies and programmes have been implemented. In developing countries, however, guidelines have been developed. In the study of a systematic review of recent developments in disaster waste management (DWM), by Zhang et al., nine aspects of planning, waste, waste treatment options, environment, economics, social considerations, organisational aspects, legal frameworks and funding were examined (Zhang et al. 2019). In the report of Asari et al., Strategies for Separation and Treatment of Disaster Waste, a manual and guidelines, were written by the Japan Society of Material Cycles and Waste Management (JSMCWM) to guide the management of the disaster waste generated from huge earthquakes and tsunamis in Japan. In compiling of guidelines for managing disaster waste, it is mentioned that a necessary task in the early recovery phase is the removal of disaster waste (e.g. rubble). Such waste can overwhelm the capacity of existing facilities and have a negative influence on other emergency response and recovery activities. At the time of a disaster, planners must determine the quantity of waste generated, gather it in temporary storage sites, and select and arrange for appropriate disposal or recycling options (Asari et al. 2013). Another important point in waste management in disaster is to pay attention to the consequences and health aspects, as in the study of Pradhananga et al., about health and safety issues in DWM. The results show that there is an adverse increase in health issues specifically for those (1) involved in DWM, (2) exposed to hazardous waste, (3) lacking health and safety guidance and training and (4) deficient in construction and engineering knowledge. Improper use of personal protective equipment (PPE) and lack of safety training has been found to be a major cause leading to health issues, where 40% of the respondents suffered from at least one health problem when involved in DWM activities (Pradhananga et al. 2021). In recent years, the holy city of Qom has faced with many fundamental changes in the demographic, cultural, social and urban development along with the increase in attention to the management of municipal services. The daily production of more than 1000 tons of municipal waste and its management is one of the most important issues in the field of municipal services. Indeed, the main purpose of this study is to examine the level of preparedness, having a contingency plan and the ability to respond urgently to waste management in disaster situations.

## Research methods and design

Qom, the capital of Qom province, is located in the boundary of the central desert of Iran (Kavir Markazi) with geographical attributes 34°38′24″N 50°52′35″E. At the 2017 census, the population of this province was 1 200 000. The present study is a cross-sectional descriptive study that was predicted and implemented in two specific phases. In the first phase of the study, an urban waste analysis programme was developed due to the lack of records of the amount of production and composition of waste in different urban areas as well as the identification of points that may be decisive in the composition of waste in times of crisis including industrial waste. The method of analysis and weighing of municipal solid waste for waste generation (tonnes per day) according to the truck counting method for 1 week per month and for all seasons were carried out (Hassanvand et al. 2008). By tools of contingency plan for decision- making in disaster and emergencies and questionnaire about resources and the current stats of waste management in the city areas (personnel, collection fleet, collection systems and methods, recycling and disposal, as well as waste composition and specifications, etc.), level of preparedness and waste management for emergency situations were assessed. This study and evaluation were performed in Qom municipal waste management office and its employees and middle managers. The reliability and validity of the questionnaire were evaluated and confirmed. In Table 1, some of the main questions and summarised questionnaire of waste management in an emergency were presented. In questions 16 and 17 of the questionnaire, 180 personnel involved in waste management were evaluated. Using DotMapper software, which is a geographic information system (GIS) software based on R software, temporary waste sites for disaster situations (map waste) were determined (Smith & Hayward 2016). Temporary waste sites selected, which are displayed in the form of green dots on the DotMapper map, based on environmental criteria such as distance, access to roads, environmental health and no damage to the ecological specification of the area, are considered. In the second phase of the study, the preliminary hazard analysis (PHA) to identify the initial hazards in the municipal waste management system and related to the earthquake scenario was considered. In this model, the final risk is calculated by semi-quantitative method and the product of the risk probability multiplied by its severity. The criteria related to the probability of risk and severity are presented according to Table 2 and Table 3. The risk assessment process was performed by considering the ethical aspects of research and non-conflict of interests as well as with the cooperation of experienced personnel of the waste management company, who thoroughly oriented the risk assessment (Tajima et al. 2014).

**TABLE 1:** Summarised questionnaire of waste management in an emergency.

Number	Summarised questions
1	Is there an emergency waste management plan in the waste management organisation?
2	Is there an emergency waste management plan in different areas of the municipality and is it available?
3	Are the steps of activating the emergency waste management plan in the waste management organisation according to the accident command system provided?
4	Is there a municipal services unit for waste management in an emergency?
5	Is a checklist of available resources (manpower, equipment required, etc.) available to evaluate emergency waste management processes?
6	Has disaster analysis been predicted and studied in municipal areas?
7	Is physical and chemical waste analysis available for each urban area?
8	Are periodic maneuvers performed regularly to assess the level of readiness of waste management services in an emergency?
9	Is the existing waste management service system capable of responding to emergencies?
10	Are there plans and temporary storage sites for emergencies in urban areas?
11	Are existing sites periodically inspected for capacity and usability?
12	Are hazardous waste production centres and units identified and specified in urban areas according to the definitions of the Waste Management Law?
13	Are these centres different from other existing centres?
14	Has the necessary provision been made to control the environmental and health pollution caused by hazardous wastes for emergencies?
15	Has the necessary provision been made for the management of the bodies, burial and identification of the dead and the relevant working groups?
16	Have personnel involved in all waste management processes received the necessary training in emergency waste management?
17	Has the necessary provision been made for the safety and protection of personnel in waste management in emergencies?

*Source:* Karunasena, G., Amartunga, D., Haigh, R. & Lill, I., 2009, ‘Post disaster waste management strategies in developing countries: Case of Sri Lanka’, *International Journal of Strategic Property Management* 13(2), 171–190. https://doi.org/10.3846/1648-715X.2009.13.171-190

**TABLE 2:** Risk assessment matrix.

Risk matrix	Severity				
	Insignificant (score 1)	Minor (score 2)	Major (score 3)	Hazardous (score 4)	Catastrophic (score 5)
**Likelihood**					
Almost certain (score 5)	5	10	**15**	**20**	**25**
Likely (score 4)	4	8	12	16	**20**
Foreseeable (score 3)	3	6	9	12	**15**
Unlikely (score 2)	2	4	6	8	10
Most unlikely (score 1)	1	2	3	4	5

*Source:* Jusoh, Z., Shattar, N.A., Majid, H.A. & Adenan, N.D., 2016, ‘Determination of Hazard in Captive Hotel Laundry Using Semi Quantitative Risk Assessment Matrix’, *Procedia-Social and Behavioral Sciences* 222, 915–922. https://doi.org/10.1016/j.sbspro.2016.05.229

**TABLE 3:** Likelihood categories and severity criteria.

Item	Definition
**Likelihood categories**	
Almost certain	Once per day
Likely	Once per week
Foreseeable	Once per month
Unlikely	Once per year
Most unlikely	Once every 5 year
**Severity criteria**	
No effect	Has no effect on health
Minor	Minor injury
Major	Injury
Hazardous	Serious or fatal injury
Catastrophic	Death

*Source:* Jusoh, Z., Shattar, N.A., Majid, H.A. & Adenan, N.D., 2016, ‘Determination of Hazard in Captive Hotel Laundry Using Semi Quantitative Risk Assessment Matrix’, *Procedia-Social and Behavioral Sciences* 222, 915–922. https://doi.org/10.1016/j.sbspro.2016.05.229

### Ethical considerations

This article followed all ethical standards for research without direct contact with human or animal subjects.

## Results

### Characteristics and quantity of municipal solid waste in disaster (earthquake scenario)

The results of municipal waste composition based on the general analysis of waste were shown in Table 4, and the amount of waste generation (tonne per day) and composition of Qom municipal waste in disaster situations (with earthquake scenario) in accordance with existing studies and records in Table 5 were presented (Brown et al. 2011; UNEP 2012).

**TABLE 4:** Municipal waste composition (%) based on the general analysis.

Textiles	Plastics and rubber	Glass	Metals	Paper and wood	Biodegradable	Construction
2.2	8.2	2.4	2.1	8.6	72.1	4.3

**TABLE 5:** Guidance and predicted values of Qom municipal waste in disaster base on population.

Number	Waste type	Component waste (%)	Waste generation (tonne per day)
1	Biodegradable waste	< 1	150†
2	Construction and demolition (concrete, brick and other building materials)	50	500‡
3	Metals	8	80‡
4	Plastics, glass, textiles and other combustible materials	12	120‡
5	Wood	7	70‡
6	Electronic components and equipment, bulky household appliances and other non-combustible materials	22	220‡

† , According to the daily per capita waste generation in disaster (150 g/d)(UNEP 2012).

‡ , Depending on the severity and extent of the disaster, waste specification and infrastructure and principles of urban planning, it varies (Brown et al. 2011; UNEP 2012).

### Determining the characteristics of municipal waste transfer sites in emergency situations (earthquake scenario)

**FIGURE 1:**
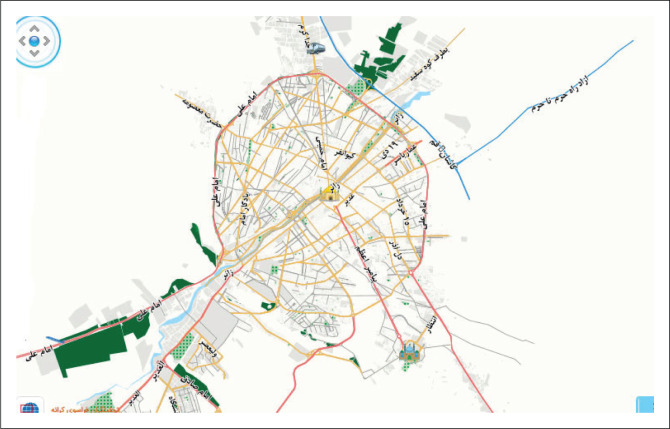
Comprehensive map of Qom city.

**FIGURE 2:**
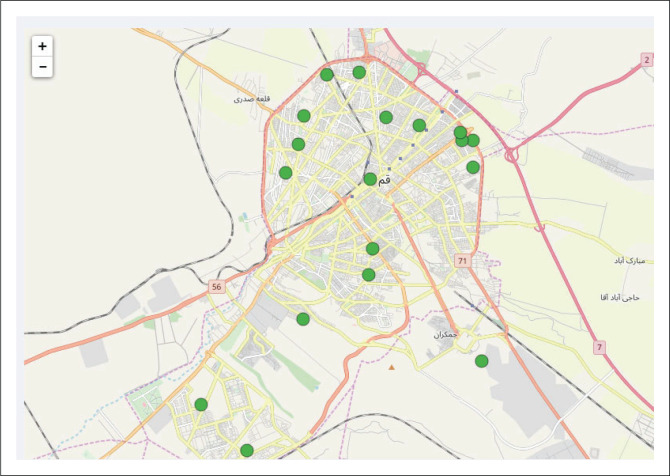
Distribution and location of waste sites in urban areas in disaster.

Based on the characteristics, the extent of the area covered by municipal services as well as the amount of municipal waste production in the areas and municipal services per population 50 000 people in the event of disaster and emergency and according to the total population of Qom number of waste transfer points (*N* = 20) as temporary waste sites for disaster situations (map waste) in urban areas based on environmental criteria were determined (Table 6, Figure 1). Distribution and location of waste sites in urban areas in disaster were shown in Figure 2.

**TABLE 6:** Number of waste transfer points in urban areas.

Urban area	Number of sites suggested
1	3
2	2
3	3
4	3
5	2
6	3
7	2
8	2

### Hazards identification and risk assessment of waste management in disaster

Hazardous events, hazard identification and risk analysis according to the method described are shown in Table 7.

**TABLE 7:** Risk analysis of waste management in disaster.

Number	Events	Hazard identification	Control of risk	Risk analysis		
				Likelihood	Severity	Risk
1	Lack of waste management plan in different urban areas	Increase the level of damage and vulnerability	Providing of contingency plan for disaster waste management	3	2	6
2	Lack of forecast of waste management unit in emergency situations in different urban areas and waste management organisation	Increasing the level of community threat, increasing the spread of pollution and environmental damage and personnel injuries	Establishment of an emergency waste management system in the municipal structure	3	4	12
3	Lack of forecasting and performing disaster and waste analysis for emergency situations in different urban areas	Failure to identify the type and composition of waste in emergency situations for each area, increased pollution due to poor management and increased damage	Planning to determine and estimate disaster waste for each urban area	3	4	12
4	Lack of temporary waste storage sites in different urban areas in emergency situations in different urban areas	Timing of waste management services in areas, increasing the level of health threats and damage to environmental infrastructure	Predicting and constructing temporary waste storage sites in different urban areas	4	4	16
5	Hazardous waste generation centres and units in urban areas have not been identified for emergencies	Increasing the level of environmental emissions of hazardous waste in the region, health aspects for the public and increasing damages and safety threats	Identification of hazardous waste generation centres in urban areas and development of emergency waste management plan	3	5	15
6	Lack of emergency waste management training program for all people involved in waste management	Safety threats and personnel protection for those involved in waste management. Threats to public health due to lack of staff awareness in controlling environmental pollution and the level of safety threats	Holding training courses for waste management staff and the general public	4	4	16

## Discussion

Physical analysis of waste in the city of Qom in Table 3 shows that most municipal waste generated under normal conditions includes 72% of biodegradable waste and recyclable waste by about 20%. Physical analysis of waste in the city of Qom for disaster and emergencies (Table 4) shows that more than 50% of the generated waste is allocated to construction waste resulting from the destruction and more than 30% to recyclable items (metals, glass, plastic, etc.). Moreover, a very small part of about 1% of biodegradable waste was determined. The rest includes household appliances and items. In this regard, the prediction of waste transfer stations for waste processing and processing facilities is the most important option (Umar et al. 2017). In the study of Omidvar et al. on sanitary waste disposal and waste management in Bam disaster after the earthquake, the highest value of waste generation is due to debris and destruction of buildings and municipal facilities in the first 72 h was determined (Omidvar, Zafari & Derakhshan 2010). Experiences from other countries, including the United States and Japan, have shown that with have pre-planned for emergency waste management, environmental health and collection of waste in damaged areas, is done quickly, however, by reducing the distance from waste production points to disposal in addition to the economic aspect of waste transportation, further damage is also prevented (Harvey et al. 2002; Reed et al. 2013; UNEP 2012). In Japan, a national report entitled ‘Post-Disaster Waste Management Experiences in the Six Cities of Ofunato, Shinomaki, Tokyo, Soma, and Sendai Miako, multi-purpose vacancies in various urban areas for emergencies as storage and processing sites were predicted’. Challenges and problems of different types of waste in each disaster, training tips for the general public and the role of waste management control systems were mentioned (Onan, Ülengin & Sennaroğlu 2015; UNEP 2012). In a systematic review study by Mark Mlike and Erica Sevill in New Zealand, technical management options in DWM, including recycling and disposal address eight key aspects of waste management in emergencies, including planning, composition and quantity of waste, treatment and environmental, economic, social, organisational and financing mechanisms, were assessed (Brown et al. 2011). Table 7 shows the results in relation to events, hazard identification and control of risk in disaster, for three events containing lack of temporary waste storage sites in different urban areas, lack of identification and determination of hazardous waste production centres in urban areas and lack of waste management training programme in emergency situations for all people involved in waste management, it should be mentioned that the results of risk analysis are in the red range, and hence, they should be considered as a main priority of the waste management organisation in disaster-related planning (Pradhananga, ElZomor & Santi Kasabdji 2021). The results of the present study are consistent with those of Asari et al. regarding the strategy for separation and treatment of disaster waste (Asari et al. 2013; Zawawi, Yusof & Ismail 2018).

Periatham and Karunasena have pointed out the basic strategies about waste management including determining the risk points, educating the community, determining possible waste and inter-sectorial coordination before the occurrence of earthquakes. This finding, in fact, confirms the results of the present study (Gabrielli et al. 2018; Jibril 2015; Karunasena et al. 2009).

## Conclusion

Developing the necessary strategies to control environmental challenges and various aspects of health outcomes in waste management is one of the basic programmes in metropolitan management. One of the issues that should be considered in pre-disaster planning is general education for the people and the community, as well as the development of specialised tests in waste-related intercity organisations. In principle, it should be noted that the issue of waste management is not limited to municipal organisations; however, social responsibility and public resilience can play an important role in waste management in disaster. Therefore, holding specialised courses for the general public and involving all organisations in order to provide the necessary resources in the field of waste management is inevitable.
